# Abdominal Lymphocele Following Anterior Lumbar Interbody Fusion: A Case Report

**DOI:** 10.7759/cureus.3357

**Published:** 2018-09-25

**Authors:** Ali Hazama, Mohamed Abouelleil, Satya Marawar, Lawrence S. Chin

**Affiliations:** 1 Neurosurgery, The State University of New York Upstate Medical University, Syracuse, USA; 2 Neurological Surgery, University of Illinois College of Medicine, Chicago, USA; 3 Orthopedic Surgery, Syracuse Veterans Affairs Hospital, Syracuse, USA

**Keywords:** anterior lumbar interbody fusion, lymphocele, retroperitoneal

## Abstract

Anterior lumbar interbody fusion (ALIF) is commonly utilized for surgical management of degenerative lumbar pathology. Although it is a reasonably safe procedure, it can potentially lead to major complications in case of neurovascular injuries. Occurrence of lymphocele after an ALIF is however rare.

We present a case of a rare abdominal lymphocele in a 56-year-old man who underwent L3-S1 ALIF and subsequently developed an abdominal lymphocele. A lymphocele can manifest in numerous ways which can affect and possibly delay diagnoses. In addition to a high index of suspicion, numerous tests such as imaging studies, fluid analysis, gram stain and culture are used to confirm the diagnosis.

Various options exist for the treatment of lymphoceles, including laparoscopic marsupialization, ultrasound-guided aspiration, sclerotherapy, peritoneal window, and external drainage. Timely diagnosis and treatment of a lymphocele results in a successful resolution in most cases.

## Introduction

Anterior lumbar interbody fusion (ALIF) is a commonly utilized modality for fusion of a wide array of lumbar degenerative pathology such as intervertebral disc degeneration, degenerative scoliosis and spondylolisthesis. It is an attractive option for the surgeon because it provides access to the entire disc space and allows a large interbody spacer that can restore lordosis and achieve high fusion rates. On account of the anterior approach it has the potential for major complications such as vascular injury, visceral damage, ureteral damage, and retrograde ejaculation. An abdominal lymphocele is a rare complication associated with this procedure [1-14]. We report the presentation and management of a lymphocele after a multilevel ALIF procedure.

## Case presentation

**Figure 1:**
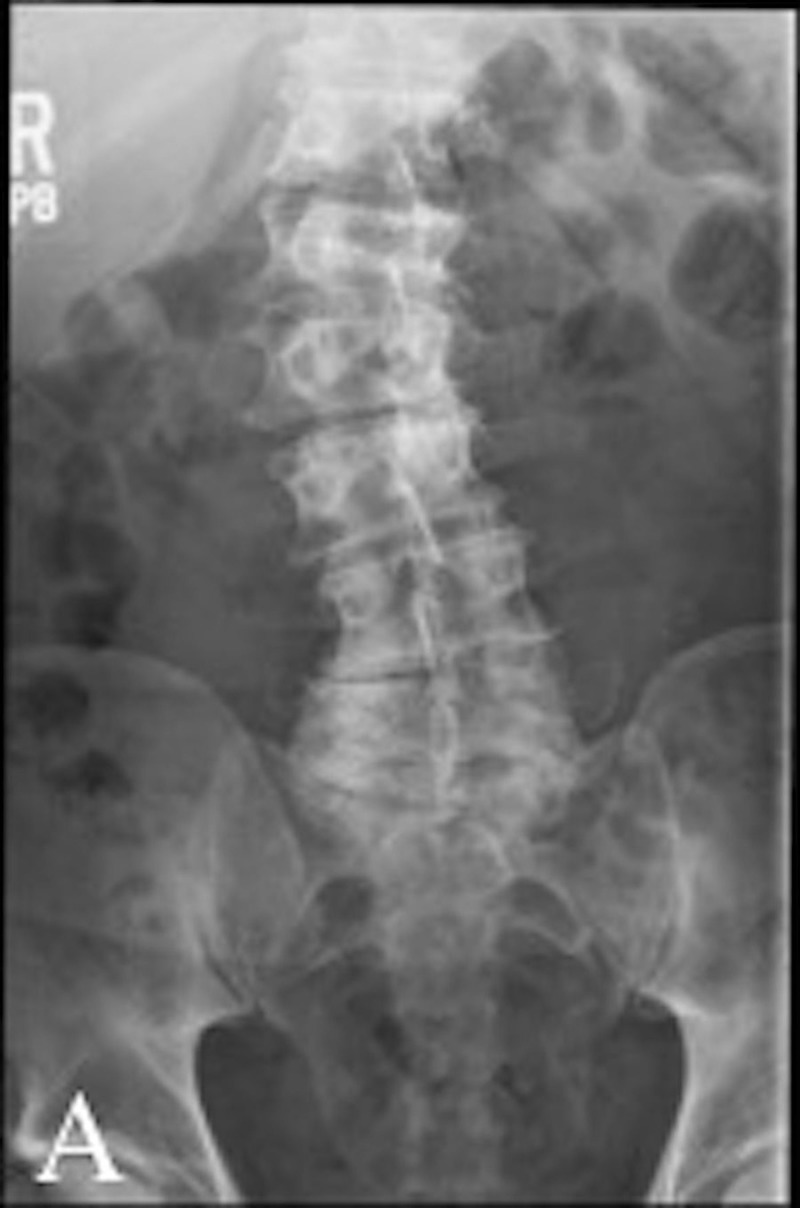
Pre-operative anterior/posterior X-rays. Pre-operative anterior/posterior X-rays demonstrating multi-level disc degeneration and neuroforaminal stenosis most severe in the lower lumbar spine.

**Figure 2:**
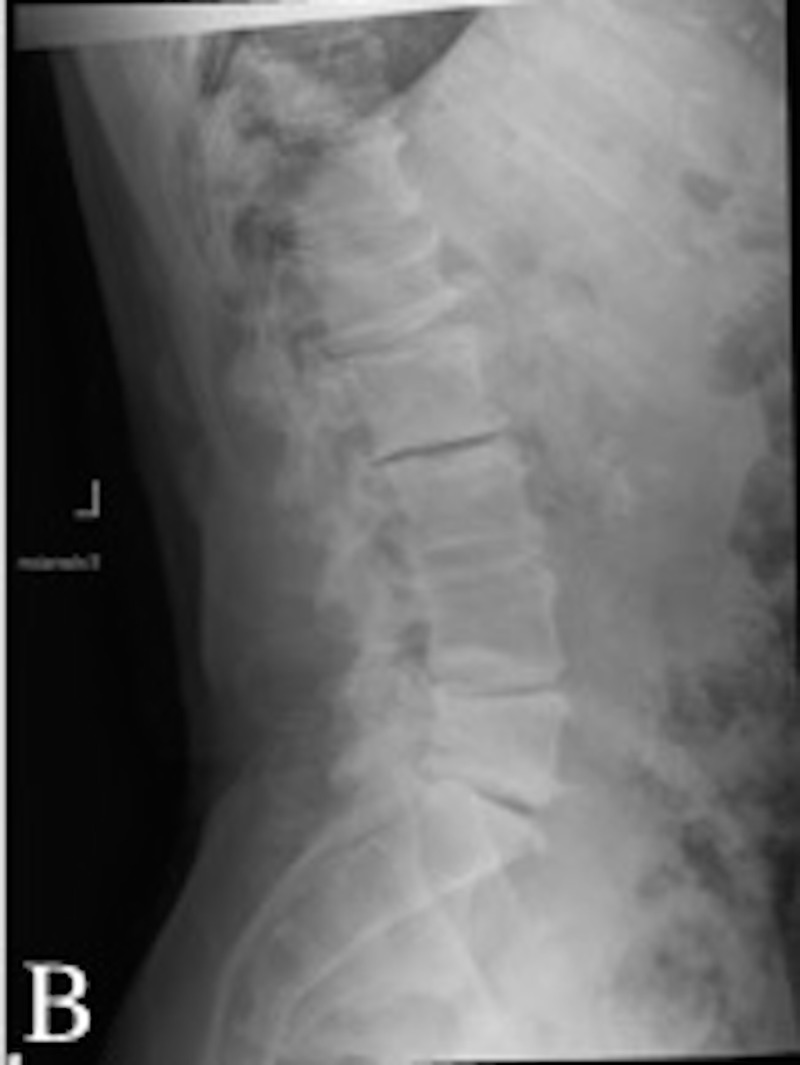
Pre-operative lateral X-rays. Pre-operative lateral X-rays degenerative scoliotic deformity, facet arthropathy, and neuroforaminal stenosis most severe in the lumbar spine.

A 56-year-old man presented to us with chronic, progressive midline low back pain associated with right lower extremity radiculopathy. The patient described the pain as radiating and achy. On the physical exam, the patient had decreased sensation in the lateral aspect of the right lower extremity. Radiological findings demonstrated multilevel central stenosis and neuroforaminal narrowing with a degenerative scoliotic deformity of the lumbar spine (Figures 1, 2). A conservative trial for up to a year with physical therapy, chiropractic and interventional pain modalities provided partial and short-lasting relief. As the pain continued to progress, the patient decided to proceed with surgery.

**Figure 3:**
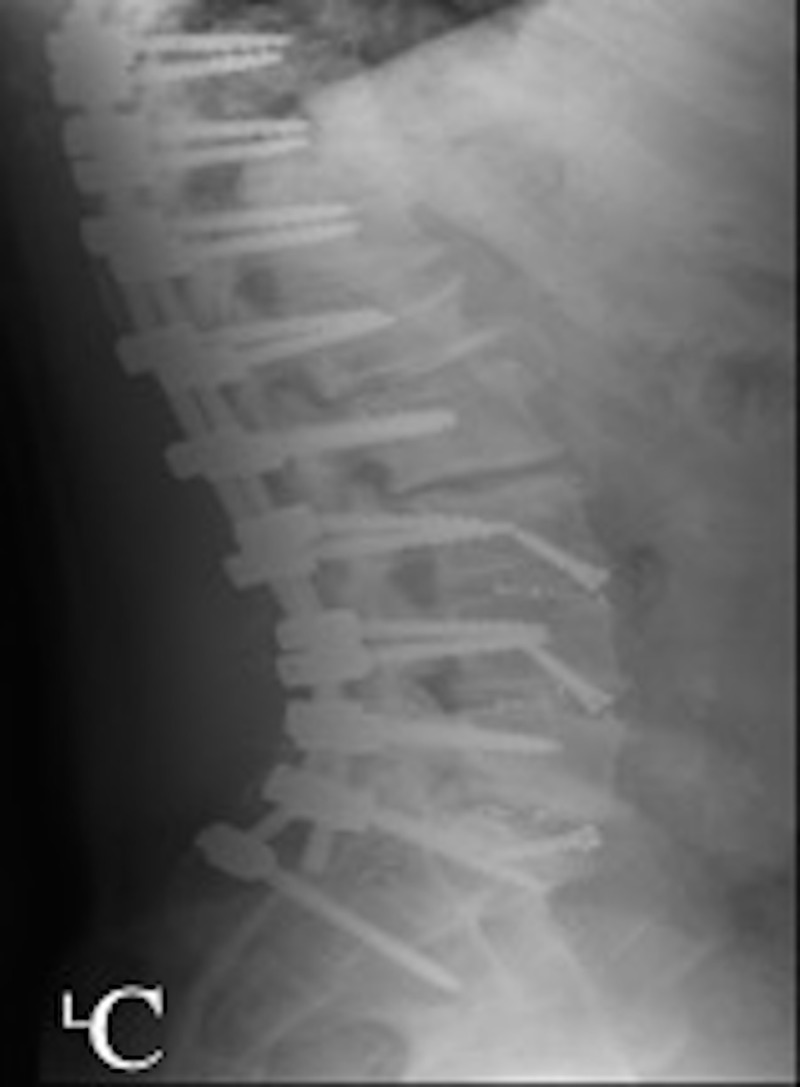
Post-operative lateral X-rays. Lateral post-operative X-ray demonstrating anterior interbody fusion cages at L3-S1. Posterior instrumentation from T10 to the pelvis is also seen.

**Figure 4:**
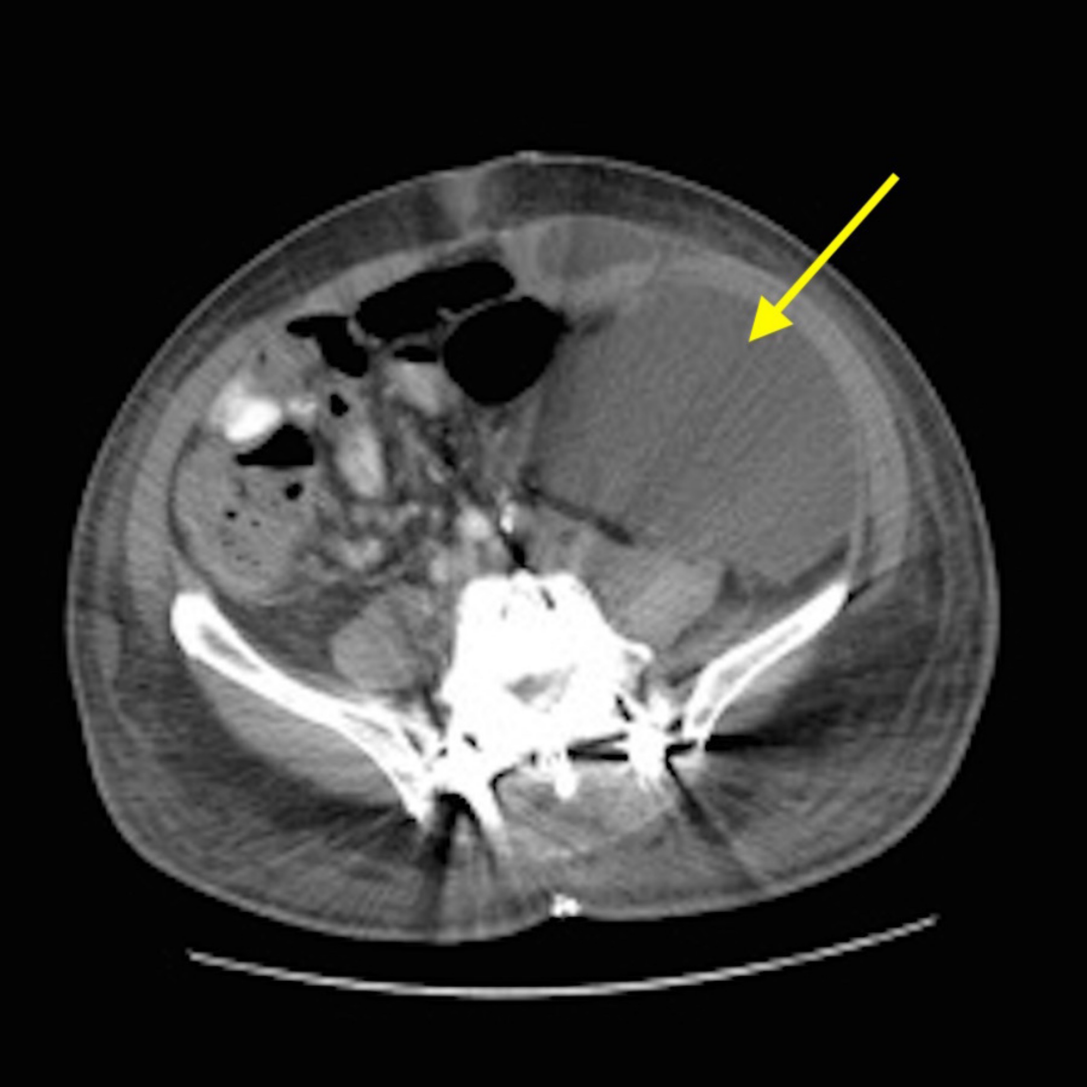
Computed tomography (CT) scan of the abdomen and pelvis. CT scan of the abdomen and pelvis demonstrating a large cystic mass in the retroperitoneal and pelvic cavities, displacing bowel rightward and compressing the left kidney.

Various surgical options were considered. Eventually the patient underwent an ALIF at the L3-S1 levels using a retroperitoneal approach followed by a T10-pelvis posterior spinal fusion and a right L4-L5 laminotomy and decompression in two stages (Figure 3). The post-operative course was complicated by abdominal distention that occurred on post-operative day eight. Computed tomography (CT) scan of the abdomen and pelvis demonstrated a large collection of retroperitoneal fluid measuring 11 x 9 x 22 cm in the left pelvis and left flank area with mass effect on the left kidney and ureter causing hydronephrosis (Figure 4). After a consult with the general surgery team and the approach surgeon, the patient underwent ultrasound-guided drainage of fluid with temporary drain placement.

The fluid was serosangionous in nature with a high output of greater than 1500 cc/day. Fluid gram stain showed no organisms and cultures also revealed no growth. He was discharged home with the drain in place for a total of 22 days after which the drain was discontinued upon clinic follow-up. The lymphocele recurred four days after the drain was removed, and the patient presented back to the emergency room with recurrence of abdominal distension. A new drain was placed again in the fluid collection, and was finally removed for paucity of drainage three weeks later. The patient’s symptoms as well as the lymphocele resolved and did not recur.

## Discussion

An abdominal lymphocele is a rare complication of anterior lumbar interbody fusion [1-15]. Presentation depends on where the damage to the lymphatic system occurs. The cisterna chyli is formed at the level of the first lumbar vertebra by lymphatic vessels from the pelvis, lower limbs, and gastrointestinal tract. Lymphatic injury proximal to the cisterna chyli results in a chyloascites or chylothorax, while injury distal to the cisterna chyli results in a lymphocele. Due to the lack of chylomicrons, in the lymphatic fluid below the cisterna chili, lymphocele presents with serous fluid with a unique yellow appearance. Chyloascites or chylothorax has a white milky appearance due to the incorporation of chylomicrons from the gastrointestinal tract [11,16].

Lymphocele accumulation in the retroperitoneal area can be asymptomatic, but may also manifest in abdominal symptoms. If leakage is constant it can cause nutritional deficiencies and can ultimately result in death with a mortality rate approaching 50% [13,14]. It is often theorized that lymphatic injury is avoided during surgery because of the decreased flow through the lymphatic system caused by dehydration resulting from the patient's fasting prior to the procedure [6]. Diagnosis of a lymphocele can be made through ultrasound and fluid analysis. The differential diagnosis of a lymphocele includes urinoma, cerebrospinal fluid leak, and ascites. Abdominal CT scan can aid in diagnosing lymphocele and ruling out urinoma, whereby the presence of fat within the lymphatic fluid causes it to become contrast enhancing [3,7]. Fluid creatinine levels are usually comparable to serum levels in a lymphocele, but are expectedly higher than serum levels in a urinoma. Fluid analysis for beta 2 transferrin can be used to rule out a cerebrospinal fluid leakage, and microbiological studies can be performed to rule out infection. Liver panel can also be assessed to rule out any ascites caused by liver damage. Although lymphangiography is the gold standard for diagnosing a lymphocele, there are complications associated with this procedure such as pulmonary embolus, skin infection, skin necrosis, and lymphatic damage due to contrast use. Benefits of doing this procedure to diagnose a lymphocele are uncertain [6,8,11].

The low incidence of symptomatic lymphoceles post ALIF can be attributed to the absorption of the leaked lymphatic fluid by collateral lymphatic vessels [6]. Consequently, most lymphoceles may be asymptomatic and are either missed or resolve spontaneously. The treatment options for clinically significant lymphoceles include laparoscopic marsupialization, ultrasound-guided aspiration, sclerotherapy, peritoneal window and external drainage. Golriz et al. found a higher recurrence rate associated with drainage via open procedure (15%) when compared to laparoscopic drainage (0–10%) [14]. The laparoscopic approach is also associated with a shorter hospital stay [9-14]. Upadhyaya et al. discussed that treating an abdominal lymphocele should be done by keeping the patient NPO (nil per os) for 7–10 days. If this fails, a drainage with fluid analysis can be done. If this additionally fails, then surgical options should be considered [14]. Our patient presented with significant clinical symptoms and we decided to perform external drainage of the lymphocele until the output declined. He did not require further surgical intervention and the lymphocele eventually resolved.

## Conclusions

Abdominal lymphocele is a rare yet significant complication that can present after an ALIF. Utilizing fluid analysis and imaging studies correctly are vital in a timely diagnosis of such a complication. While most retroperitoneal lymphoceles are asymptomatic and resolve spontaneously, some present with significant symptoms and require drainage or surgical intervention. There are many ways to approach treatment of an abdominal lymphocele that vary due to a surgeon’s preference. While this does not manifest in any long-term complications, it is a rare acute complication post ALIF that surgeons should be aware of.
